# Retraction Note: Prophylactic treatment with transdermal deferoxamine mitigates radiation-induced skin fibrosis

**DOI:** 10.1038/s41598-025-00263-4

**Published:** 2025-05-12

**Authors:** Abra H. Shen, Mimi R. Borrelli, Sandeep Adem, Nestor M. Diaz Deleon, Ronak A. Patel, Shamik Mascharak, Sara J. Yen, Blake Y. Sun, Walter L. Taylor, Michael Januszyk, Dung H. Nguyen, Arash Momeni, Geoffrey C. Gurtner, Michael T. Longaker, Derrick C. Wan

**Affiliations:** 1 Hagey Laboratory for Pediatric Regenerative Medicine, Division of Plastic Surgery, Department of Surgery, Stanford University School of Medicine, 257 Campus Drive, Stanford, CA 94305-5148 USA; 2 Stanford Institute for Stem Cell Biology and Regenerative Medicine, Stanford University School of Medicine, Stanford, CA USA

Retraction of: *Scientific Reports* 10.1038/s41598-020-69293-4, published online 23 July 2020

The Editors have retracted this Article. Two images in Figure 3b appear to overlap, and within Figure 3e, panels IR no DFO and DFO ppx partially overlap. The Authors were unable to provide the raw data and original images for this article upon request. The Editors have lost confidence in the data and conclusions of this article.

Derrick C. Wan disagrees with this retraction. All other Authors did not respond to correspondence from the Editors regarding this retraction.

